# Opioid and Neuropathic Pain Medication Use After ACDF for Degenerative Cervical Spine Disease—Nationwide FinSpine Register Study

**DOI:** 10.1097/BRS.0000000000005705

**Published:** 2026-04-09

**Authors:** Nikolai Klimko, Nils Danner, Laura Schildt, Henri Salo, Ville Leinonen, Jukka Huttunen

**Affiliations:** a Department of Neurosurgery, Kuopio University Hospital and Institute of Clinical Medicine, University of Eastern Finland, Kuopio, FI; b Finnish Institute for Health and Welfare, Helsinki, FI

**Keywords:** ACDF, degenerative cervical spine disease, FinSpine, opioid use, new strong-opioid starter, neuropathic pain medication

## Abstract

**Study Design.:**

Longitudinal, nationwide register study.

**Objective.:**

To examine the use of opioids and neuropathic pain medications after primary anterior cervical discectomy and fusion (ACDF) for degenerative cervical spine disease (DCSD), and to identify independent factors associated with postoperative use.

**Summary of Background Data.:**

ACDF is used for cervical radiculopathy and myelopathy, yet many patients continue analgesic use postoperatively. Prolonged opioid use is associated with dependence, poorer clinical outcomes, and reduced return-to-work rates. Evidence on postoperative medication trajectories and their predictors in population-based cohorts remains limited.

**Materials and Methods.:**

Data were obtained from the nationwide FinSpine register and the Finnish prescription database, which records all outpatient prescription drug purchases. Consecutive patients undergoing primary ACDF for DCSD between 2017 and 2022 were included. Repeated purchases were defined as at least two purchases of the same drug during months 2 to 12 postoperatively. New strong-opioid users were defined as patients who were strong-opioid naïve six months preceding surgery and met the repeated postoperative purchase criterion. Multivariable logistic regression was used to identify independently associated covariates.

**Results.:**

The cohort included 4366 patients. Preoperatively, 41.9% (n=1830) purchased opioids and 41.2% (n=1798) gabapentinoids. Repeated postoperative purchases were observed in 16.5% for opioids and 15.6% for gabapentinoids. Among preoperative opioid and gabapentinoid users, cessation rates were 69.5% and 70.9%, respectively. In previously strong-opioid naïve patients, the incidence of new repeated strong-opioid purchases was 2.2% after ACDF. Independent predictors of repeated postoperative purchases included preoperative pain duration of more than one year, smoking, higher baseline NDI, central canal stenosis, adverse working status, and preoperative purchases of the same drug class.

**Conclusion.:**

A considerable proportion of patients continued to purchase opioids and gabapentinoids during the first postoperative year after ACDF. New strong-opioid initiation postoperatively was uncommon. Nationwide prescription data offer an objective and complete measure of postoperative medication dispensing for outcome assessment.

Anterior cervical discectomy and fusion (ACDF) is a common surgical treatment for degenerative cervical spine pathologies, which often provides significant relief of radiculopathy and neck pain.^1–4^ However, a considerable subset of patients experience persistent or recurrent symptoms after surgery, and a notable proportion continue to use analgesic medications long after the acute perioperative phase.^2,3,5,6^ Nonsteroidal anti-inflammatory drugs (NSAIDs), paracetamol, and gabapentinoids are supported by robust evidence and are widely used as part of multimodal pain management.^7,8^ In contrast, prolonged use of opioids is associated with several problems, including dependence, poorer clinical outcomes, and reduced return-to-work rates.^9–13^

Given the clinical and public health implications of prolonged medication use after ACDF, evaluating postoperative trajectories of opioid and neuropathic pain medication use is essential for assessing outcomes. Existing data in cervical spine surgery remain limited, as previous studies have relied on selected patient populations or smaller cohorts, and postoperative pain medication use has not been assessed in a nationwide setting.^5,6,14^

The aim of this study was to investigate repeated postoperative purchases of opioids and neuropathic pain medications after ACDF and to identify factors associated with continued postoperative use by using a nationwide spine surgery register linked with a nationwide prescription database.

## MATERIALS AND METHODS

### FinSpine Register

FinSpine is the Finnish national quality register for spine surgery, administered by the Finnish Institute for Health and Welfare.^15^ The tax-funded public healthcare system provides equal access to medical care without socioeconomic selection, ensuring that services are available across the entire population. Since its inception in 2016, the register has accumulated procedure-specific data—such as surgery type, diagnosis for the surgery, and perioperative complications—entered by spine surgeons; patient-reported outcome and experience measures collected at defined time points; and selected information obtained directly from electronic health records.^15^ All 23 public hospitals and major private centers performing spine surgery in Finland contribute to FinSpine, ensuring complete national coverage.^15^

### Prescription Data

Prescription data were obtained from the nationwide prescription database maintained by the Social Insurance Institution of Finland, which records all outpatient prescription drug purchases made in Finland. These data were then linked with the FinSpine register records at the individual level.

For descriptive pooling of purchased quantities across medications, we standardized dispensed amounts using the WHO Defined Daily Dose (DDD) methodology, which represents the assumed average maintenance dose per day for a drug’s principal indication in adults.^16^ For codeine, no DDD exists for analgesic use; therefore, a statistical DDD of 240 mg was applied, based on the dosing guideline of the European Medicines Agency and consistent with established Finnish pharmacological practice.^17,18^

### Study Setting and Patient Cohort

**Figure 1:**
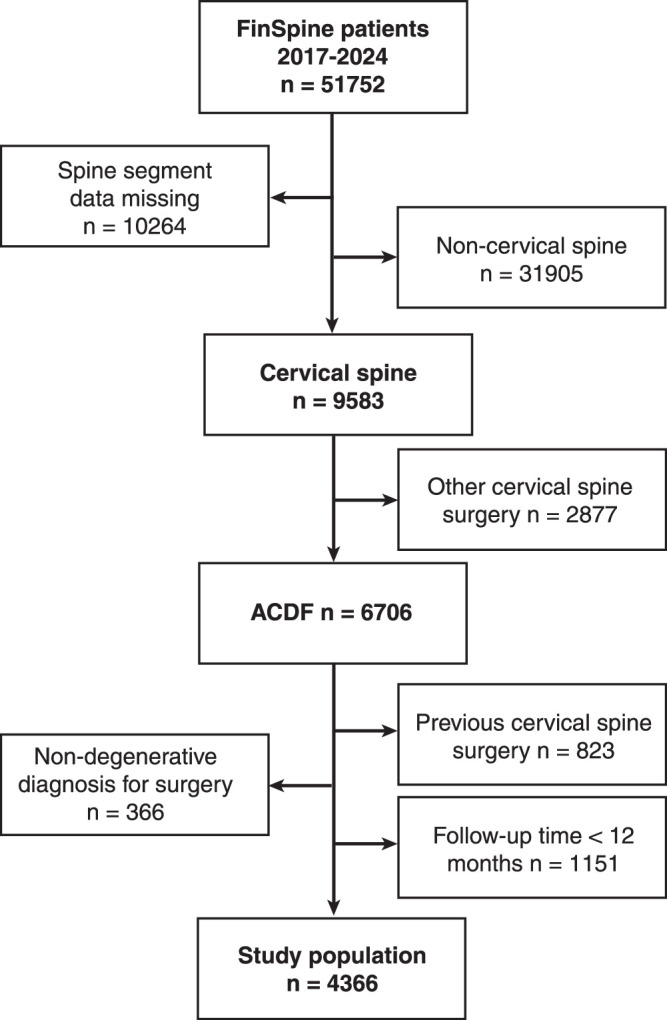
Flowchart of the FinSpine register study population. The study cohort consists of consecutive patients who underwent ACDF surgery for degenerative cervical spine disease between January 1, 2017, and November 30, 2022.

This longitudinal register-based study utilized two prospectively collected nationwide data sets. The cohort was derived from the FinSpine register; a patient inclusion flowchart is shown in Figure 1. We included consecutive patients undergoing primary ACDF for degenerative cervical spine disease, without prior cervical spine surgery. Procedures were limited to those performed between January 1, 2017, and November 30, 2022, to ensure at least 12 months’ postoperative follow-up, as prescription data were available through November 30, 2023, at the time of analysis.

### Defining Repeated Postoperative Drug Purchases

Repeated postoperative drug purchases were defined as at least two purchases of the same drug between 2 and 12 months (31–365 d) after ACDF. Purchases made within the first postoperative month were excluded to avoid capturing perioperative analgesics prescribed for immediate postoperative pain.

### New Strong-Opioid Users

New strong-opioid users were defined to examine new-onset postoperative strong-opioid initiation. Strong opioids include oxycodone and buprenorphine. Patients were classified as new strong-opioid users if they were strong-opioid naïve for at least six months preoperatively and met the study definition for repeated postoperative strong-opioid purchases during months 2 to 12 after ACDF.

### Potential Covariates for Repeated Postoperative Purchases

Potential covariates were selected from variables routinely collected in FinSpine. In addition, dichotomized baseline variables identified in our previous study of the same study population as independent predictors of 12-month clinical outcome after primary ACDF were included.^3^

The following potential covariates were entered into the multivariable regression models: age, sex, smoking status, baseline Neck Disability Index (≤42 *vs.* >42), baseline VAS arm and neck pain scores, duration of symptoms (≤1 y *vs.* >1 y), diagnosis category, perioperative complication, preoperative working status, and preoperative use of pain medications [NSAIDs, paracetamol, muscle relaxants, gabapentinoids, tricyclic antidepressants (TCAs), serotonin-norepinephrine reuptake inhibitors (SNRIs), tramadol, codeine, buprenorphine, and oxycodone]. Models were handled as complete-case analyses, and complete data necessary for modeling were available for 1770 (40.8%) patients.

### Statistical Analyses

All analyses were performed in R (version 4.4.1). Normality was evaluated using the Shapiro-Wilk test and visual inspection of Q–Q plots (Supplemental Figure, Supplementary Digital Content 1, http://links.lww.com/BRS/D65). Parametric continuous variables were analyzed with the independent *t* test, and nonparametric variables with the Wilcoxon rank-sum test. Categorical variables were compared using the χ^2^ test, or Fisher exact test when expected cell counts were <5. Descriptive statistics were reported as means with SDs for continuous variables and as counts with percentages for categorical variables.

Logistic regression models using Firth penalized likelihood were applied to account for low event frequencies. Model performance, including discrimination and calibration, was assessed using internal validation with bootstrapping (500 resamples) and is reported in the Supplemental Table, Supplementary Digital Content 2, http://links.lww.com/BRS/D66. Separate models were constructed for the subgroup of new strong-opioid users and for selected drug groups: oxycodone (OXY), buprenorphine (BUP), tramadol (TRA), codeine (COD), and gabapentinoids (GBP). Drug groups were selected based on observed purchasing patterns, clinical relevance for degenerative cervical spine disease, and alignment with national and international recommendations.^7,17,19^

### Ethics Approval

This register study has been permitted by the Finnish Institute for Health and Welfare and approved by the Ministry of Social Affairs and Health of Finland (THL/216/6.02.00/2024). According to Finnish research legislation, register-based studies do not require separate institutional ethical board approval.

## RESULTS

A total of 4366 consecutive patients underwent primary ACDF for degenerative cervical spine disease between January 2017 and November 2022. The mean age was 52.7 years (SD: 10.9), and 47.6% were female. The mean baseline NDI was 42.8 (SD: 17.2). Mean VAS scores were 56.2 (SD: 27.6) for arm pain and 54.7 (SD: 27.6) for neck pain. Pain duration exceeded one year in 46.0% of patients, and 24.7% were active smokers. Most surgeries were single-level fusions (68.5%), and standalone cages were used in 98.7% of cases.

**Figure 2:**
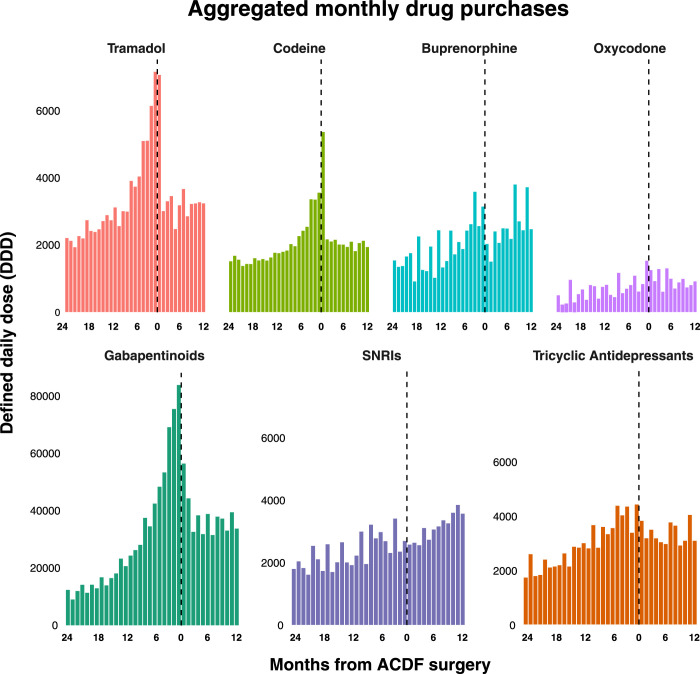
Aggregated monthly dispensing of medications in the cohort in relation to ACDF. Units are displayed in defined daily doses (DDD) of dispensed amounts. SNRIs indicate serotonin-norepinephrine reuptake inhibitors.

Prescription data analysis identified 52,954 purchases of opioids and 48,896 purchases of neuropathic pain medications (gabapentinoids, TCAs, and SNRIs) made by the study population between January 2014 and November 2023. Aggregated monthly amounts of drugs purchased in relation to the time of surgery are presented in Figure 2.

Drug purchase and postoperative cessation rates are summarized in Table 1. Preoperatively, 41.9% (n=1830) of patients purchased opioids, and 41.2% (n=1798) purchased gabapentinoids within six months before ACDF. Repeated postoperative purchases occurred in 16.5% (n=721) for opioids and 15.6% (n=681) for gabapentinoids. Among preoperative users, postoperative cessation rates were 69.5% for opioids and 70.9% for gabapentinoids; oxycodone had the highest cessation rate at 78.4%.

**TABLE 1:** Drug Purchases and Postoperative Cessation Rates

Drug Group	Preoperative Purchases, n (%)	Postoperative Repeated Purchases, n (%)	Postoperative Cessation, n (%)
Oxycodone	278 (6.4)	126 (2.9)	218 (78.4)
Buprenorphine	114 (2.6)	85 (2.0)	76 (66.7)
Tramadol	894 (20.5)	286 (6.5)	697 (78.0)
Codeine	1029 (23.6)	358 (8.2)	788 (76.6)
Gabapentinoids	1798 (41.2)	681 (15.6)	1275 (70.9)
SNRIs	138 (3.2)	136 (3.1)	56 (40.6)
Tricyclic antidepressants	667 (15.3)	330 (7.6)	454 (68.1)
All opioids	1830 (41.9)	721 (16.5)	1271 (69.5)
Strong opioids	375 (8.6)	201 (4.6)	269 (71.7)
Weak opioids	1674 (38.3)	592 (13.6)	1182 (70.6)

Postoperative cessation rates represent patients who purchased the drug preoperatively but had no repeated postoperative purchases. For weak opioids, cessation was defined as the absence of repeated postoperative purchases of the same drug or any stronger opioid.

SNRIs indicate serotonin–norepinephrine reuptake inhibitors.

**Figure 3:**
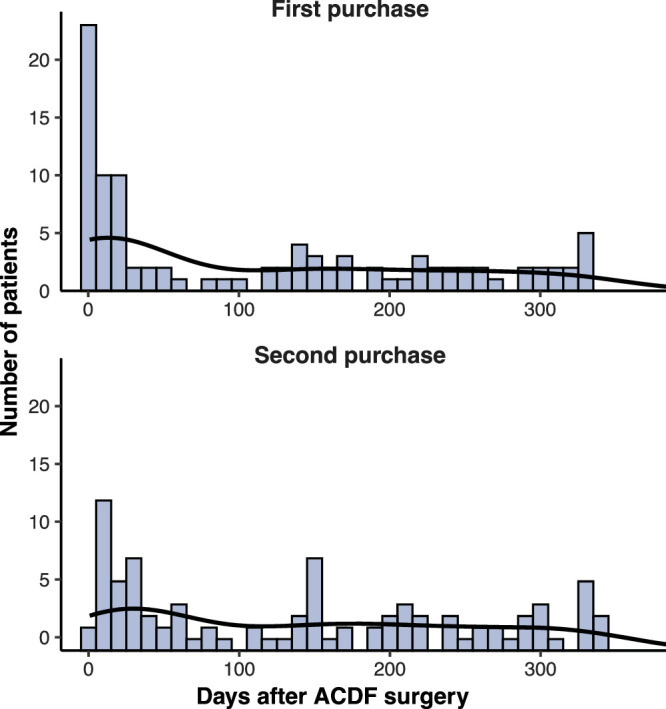
Timing of postoperative strong-opioid purchases among preoperatively strong-opioid naïve patients. Histograms show postoperative strong-opioid purchases in 10-day bins during the first postoperative year; density curves are overlaid. The subgroup met the repeated postoperative purchases definition (≥2 strong-opioid purchases during months 2 to 12 after ACDF); purchases within 30 days are shown for timing but were not included in the repeated purchases definition.

**Figure 4:**
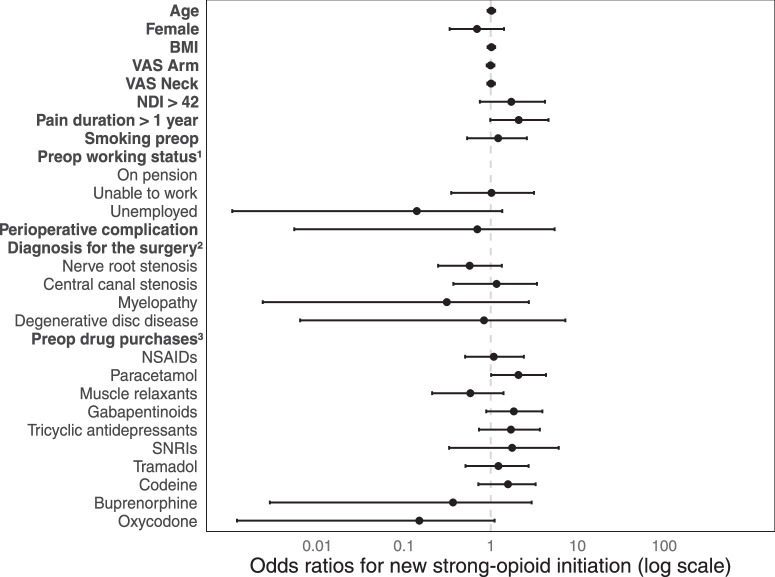
Forest plot displaying adjusted odds ratios with 95% CIs from the logistic regression model for new strong-opioid users during months 2 to 12 after ACDF. All patient-reported outcome measures represent preoperative values. Strong opioids were defined as buprenorphine and oxycodone. ^1^For preoperative working status, “working” is the reference category. ^2^For diagnosis, patients undergoing surgery for disc herniation served as the reference category. ^3^Preoperative drug purchases refer to purchases made within the six months preceding surgery. Statistical significance was defined as *P*<0.05. BMI indicates body mass index; NDI, Neck Disability Index; NSAIDs, nonsteroidal anti-inflammatory drugs; SNRIs, serotonin–norepinephrine reuptake inhibitors; VAS, visual analog scale.

Ninety-five patients (2.2%) met the definition of new strong-opioid users. Of these, 23.2% (n=22) made their first purchase within 30 days after ACDF, in addition to the purchases required to meet the subgroup criterion of at least two purchases during months 2 to 12. The distribution of first and second purchases is shown in Figure 3. During the first postoperative year, the number of strong-opioid purchases per patient ranged from 2 to 23, with a median of 4 (IQR=4.5). In univariable analyses, differences were observed between this group and the remainder of the cohort (Table 2). In multivariable modeling, no independently associated factors were identified (Figure 4).

**TABLE 2:** Baseline Characteristics and Subgroup Comparisons of the Study Population

Variable	Total	Missing, n (%)	New Strong-Opioid Starters, n (%)	Remaining Cohort, n (%)	*P*
Patients	4366	0	95	4271	
Age, mean±SD	52.7±10.9	0	55.44±13.11	52.67±10.83	0.043
Female	2080 (47.6)	0	45 (47.4)	2035 (47.6)	—
BMI, mean±SD	28.5±5.3	2102 (48.1)	29.38±9.13	28.46±5.21	—
VAS arm (0–100)	56.2±27.6	2230 (51.1)	60.91±25.40	56.08±27.68	—
VAS neck (0–100)	54.7±27.6	2174 (49.8)	61.39±26.83	54.58±27.64	—
NDI (0–100)	42.8±17.2	2197 (50.3)	53.68±15.05	42.62±17.21	<0.001
NDI >42	1087 (50.1)	2197 (50.3)	31 (75.6)	1056 (49.6)	0.001
Pain duration		2129 (48.7)			
3–12 mo	904 (40.4)		17 (38.6)	887 (40.4)	—
6–12 wk	205 (9.2)		1 (2.3)	204 (9.3)	—
<6 wk	99 (4.4)		0	99 (4.5)	—
>1 yr	1029 (46.0)		26 (59.1)	1003 (45.7)	—
Smoking preoperatively	560 (24.7)	2100 (48.1)	14 (31.8)	546 (24.6)	—
Preoperative working status		2114 (48.4)			
Working	1203 (53.4)		12 (29.3)	1191 (53.9)	0.006
Unable to work	567 (25.2)		16 (39.0)	551 (24.9)	0.006
On pension	337 (15.0)		11 (26.8)	326 (14.7)	0.006
Unemployed	145 (6.4)		2 (4.9)	143 (6.5)	0.006
Diagnosis for the surgery		0			
Disc herniation	1420 (32.5)		29 (30.5)	1391 (32.6)	—
Nerve root stenosis	2153 (49.3)		39 (41.1)	2114 (49.5)	—
Central canal stenosis	548 (12.6)		19 (20.0)	529 (12.4)	—
Myelopathy	169 (3.9)		6 (6.3)	163 (3.8)	—
Degenerative disc disease	76 (1.7)		2 (2.1)	74 (1.7)	—
Radiculopathy	1085 (25.3)	77 (1.8)	25 (27.2)	1060 (25.3)	—
SCI	427 (9.9)	69 (1.6)	16 (17.0)	411 (9.8)	0.020
No. levels operated		2232 (51.1)			
1	1462 (68.5)		30 (61.2)	1432 (68.7)	—
2	620 (29.1)		19 (38.8)	601 (28.8)	—
3	50 (2.3)		0	50 (2.4)	—
4	2 (0.1)		0	2 (0.1)	—
Operated level		2233 (51.1)			
C3-4	94 (3.3)		4 (5.9)	90 (3.2)	—
C4-5	346 (12.1)		9 (13.2)	337 (12.1)	—
C5-6	1278 (44.7)		33 (48.5)	1245 (44.6)	—
C6-7	1036 (36.2)		18 (26.5)	1018 (36.5)	—
C7-Th1	104 (3.6)		4 (5.9)	100 (3.6)	—
Type of fixation		0			
Standalone cage	4311 (98.7)		93 (97.9)	4218 (98.8)	—
Cage and plate	55 (1.3)		2 (2.1)	53 (1.2)	—
Perioperative complication	77 (1.7)	107 (2.5)	—	—	—
Preoperative drug purchases					
NSAIDs	2589 (59.3)	0	60 (63.2)	2529 (59.2)	—
Paracetamol	1424 (32.6)	0	41 (43.2)	1383 (32.4)	0.027
Muscle relaxants	1065 (24.4)	0	17 (17.9)	1048 (24.5)	—
Gabapentinoids	1798 (41.2)	0	44 (46.3)	1754 (41.1)	—
Tricyclic antidepressants	667 (15.3)	0	21 (22.1)	646 (15.1)	—
SNRIs	138 (3.2)	0	4 (4.2)	134 (3.1)	—
Tramadol	894 (20.5)	0	27 (28.4)	867 (20.3)	—
Codeine	1029 (23.6)	0	35 (36.8)	994 (23.3)	0.002
Buprenorphine	114 (2.6)	0	0	114 (2.7)	—
Oxycodone	278 (6.4)	0	0	278 (6.5)	—
Any opioids	1830 (41.9)	0	53 (55.8)	1777 (41.6)	0.006
Any neuropathic pain drug	2228 (51.0)	0	56 (58.9)	2172 (50.9)	—
Any repeated postop opioid purchases	721 (16.5)	0	95 (100)	652 (15.3)	<0.001
Repeated postop gabapentinoid purchases	681 (15.6)	0	47 (49.5)	634 (14.9)	<0.001

Parametric continuous variables were analyzed with independent *t* tests and nonparametric variables with Wilcoxon rank-sum tests. Categorical variables were analyzed with χ^2^ or Fisher exact tests, as appropriate.

BMI indicates body mass index; NDI, Neck Disability Index; NSAIDs, nonsteroidal anti-inflammatory drugs; SCI, spinal cord injury; SNRIs, serotonin–norepinephrine reuptake inhibitors; VAS, visual analog scale.

Significant *P*<0.05 is reported.

In the whole cohort, median timings and corresponding IQRs for first purchases were: OXY −1.3 months (33.8), BUP −6.6 months (53.8), TRA −9.5 months (43.4), COD −34.5 months (57.0), and GBP −5.8 months (24.7) before surgery. Last purchases were concentrated shortly after surgery, with medians of: OXY 2.3 months (31.8), BUP 4.6 months (44.3), TRA 0.0 months (29.2), COD 0.1 months (35.8), and GBP 3.4 months (29.9) (Supplemental Figure, Supplementary Digital Content 3, http://links.lww.com/BRS/D67).

**Figure 5:**
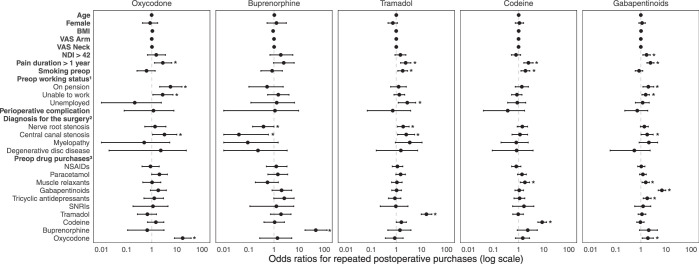
Forest plots displaying adjusted odds ratios with 95% CIs from the logistic regression models for repeated postoperative drug purchases (2–12 months after ACDF). Separate models are shown for oxycodone, buprenorphine, tramadol, codeine, and gabapentinoids. All patient-reported outcome measures represent preoperative values. ^1^For preoperative working status, “working” is the reference category. ^2^For diagnosis, patients undergoing surgery for disc herniation served as the reference category. ^3^Preoperative drug purchases refer to purchases made within the six months preceding surgery. *P<0.05. BMI indicates body mass index; NDI, Neck Disability Index; NSAIDs, nonsteroidal anti-inflammatory drugs; SNRIs, serotonin–norepinephrine reuptake inhibitors; VAS, visual analog scale.

In multivariable regression models for repeated postoperative purchases (Figure 5), pain duration greater than one year was associated with repeated OXY [OR=2.76 (1.29–6.24), *P*=0.009], TRA [OR=2.36 (1.48–3.79), *P*<0.001], COD [OR=2.47 (1.63–3.76), *P*<0.001] and GBP [OR=2.42 (1.76–3.33), *P*<0.001] purchases, but not BUP. Preoperative smoking was associated with repeated TRA [OR=1.80 (1.15–2.79), *P*=0.010] and COD [OR=1.88 (1.26–2.79), *P*=0.002] purchases, and baseline NDI >42 with repeated GBP purchases [OR=1.69 (1.21–2.37), *P*=0.002]. Compared with disc herniation, central canal stenosis was associated with repeated OXY [OR=3.21 (1.08–9.96), *P*=0.036], TRA [OR=2.49 (1.13–5.36), *P*=0.024], and GBP [OR=1.79 (1.02–3.09), *P*=0.041] purchases.

Working status was associated with medication purchases: patients on pension and those unable to work had higher odds of repeated OXY [OR=5.57 (2.07–16.01), *P*=0.001; OR=2.72 (1.11–7.01), *P*=0.028] and GBP [OR=1.99 (1.22–3.25), *P*=0.006; OR=1.56 (1.11–2.20), *P*=0.011] purchases, while unemployment was associated with repeated TRA purchases [OR=2.74 (1.20–5.90), *P*=0.016].

Preoperative purchases of the same drug class showed the strongest associations with postoperative recurrence, most notably for OXY [OR=17.02 (8.05–36.99), *P*<0.001]. No associations were observed for age, sex, BMI, VAS arm, VAS neck, or perioperative complications. Internally validated model performance metrics are provided in Supplemental Table, Supplemental Digital Content 2, http://links.lww.com/BRS/D66; optimism-corrected AUC ranged from 0.71 to 0.85.

## DISCUSSION

This nationwide study examined repeated postoperative purchases of opioids and neuropathic pain medications after primary ACDF for degenerative cervical spine disease. Postoperatively, repeated purchases occurred in 16.5% for opioids and 15.6% for gabapentinoids. Preoperative pain duration >1 year, smoking, higher baseline NDI, surgery for central canal stenosis, inability to work or pension status, and preoperative drug purchases were independently associated with repeated postoperative purchases across several drug groups.

Purchase trajectories showed that the use of weak opioids and gabapentinoids increased in the months preceding ACDF and declined rapidly thereafter, mirroring expected improvement in cervical radiculopathy and neck pain following surgery. A marked postoperative spike in weak-opioid purchases likely reflects prescriptions for the acute postoperative pain phase after discharge. In contrast, buprenorphine and oxycodone showed no clear perioperative shift, although oxycodone exhibited the highest postoperative cessation rate. SNRIs and tricyclic antidepressants showed minimal perioperative change, potentially reflecting their wider therapeutic use across indications not specific to the surgical episode.^19–22^

A total of 95 patients, representing 2.2% of the cohort, were strong-opioid naïve preoperatively and had repeated postoperative purchases of strong opioids. In univariable analyses, this subgroup differed from the remainder of the cohort, but no independently associated factors were identified in multivariable modeling, which may be explained by the limited number of events. The low incidence of new strong-opioid use, together with the high cessation rates among preoperative oxycodone users, suggests that ACDF is unlikely to provoke either new or continued sustained strong-opioid consumption. Accordingly, the use of strong opioids is more likely related to factors other than ACDF or the underlying degenerative cervical pathology. For context, a recent nationwide Austrian study examining new persistent opioid use after surgery identified spinal surgery as an independent risk factor and reported an incidence of 6.8%.^23^

Long preoperative pain duration, exceeding one year, was among the strongest independently associated factors for continued postoperative use of opioids and gabapentinoids. A longer pain history extends the period during which analgesic medications may be needed, increasing cumulative exposure and potentially contributing to continued use after surgery. In earlier FinSpine analyses, pain lasting more than one year was the strongest predictor of poorer patient-reported outcomes.^3^ Higher baseline NDI scores showed the same pattern, being independently associated with continued gabapentinoid use in this study and with worse outcomes in a prior FinSpine study.^3^

Smoking has been linked to increased postoperative opioid use in several spine surgery cohorts, including ACDF and lumbar procedures.^5,24,25^ In the present study, smoking remained an independently associated factor for continued postoperative use of weak opioids, specifically codeine and tramadol. This association may reflect behavioral factors related to dependency, but smoking has also been associated with poorer outcomes and reduced fusion rates after ACDF.^3,26,27^

Preoperative work status demonstrated associations with postoperative medication use. Patients who were on a pension or unable to work had higher odds of continued use of opioids and gabapentinoids. These categories include individuals on long-term sick leave or disability pension, and those receiving old-age pension, representing groups with generally higher illness burdens. In contrast, unemployment was associated only with slightly higher odds of continued tramadol use and likely reflects the heterogeneity of this group in terms of medical background and general health.

The initial diagnosis, that is, the indication for surgery, influenced postoperative drug purchases. Patients undergoing ACDF for central canal stenosis had higher odds of postoperative use of oxycodone, tramadol, and gabapentinoids than those treated for disc herniation. Central canal stenosis often reflects a more advanced degenerative process with longer symptom duration and greater baseline disability, which may partly account for the increased likelihood of continued postoperative analgesic use in this group.

Although no association was found between perioperative complications and postoperative use of any drugs in the present cohort, an iatrogenic contribution cannot be fully excluded. Perioperative complications are frequently underreported in surgeon-reported register data, and persistent pain arising from an iatrogenic nerve root injury may only become clinically evident later in recovery.^28–30^ This may contribute to noise in the interpretation of postoperative pain medication purchasing patterns.

Preoperative opioid exposure is well established as a predictor of postoperative continuation, as shown across lumbar and cervical cohorts.^5,14,24,25,31–35^ Much of the existing evidence is derived from data sets in which the clinical indication for each prescription is not available, a limitation also underscored in a recent systematic review of opioid use and outcomes after spine surgery.^36^ The present study shares this limitation, because the Finnish prescription database does not record prescribing indication. Even so, three features of our data support interpretation within the context of degenerative cervical pathology. Purchases of weak opioids and gabapentinoids increased in the months preceding ACDF and declined promptly thereafter. Cessation rates exceeded 70% for gabapentinoids and for all opioids except buprenorphine. Furthermore, the timing of first and last purchases for most drugs was concentrated around the perioperative period. These patterns suggest that a substantial proportion of analgesic use in this cohort was driven by cervical symptoms. Nevertheless, some of the baseline use undoubtedly reflects unrelated conditions and remains an unavoidable source of confounding when evaluating preoperative drug use as a predictor of postoperative continuation.

This study has several notable strengths. Finnish nationwide prescription data provide an objective measure of postoperative medication use, using purchases as a proxy for use and cessation. The prescription register captures all outpatient prescription drug purchases, ensuring complete coverage. Opioid agents were analyzed separately, avoiding distortions from dose-conversion methods.^37–39^ Linkage with FinSpine, a nationwide surgical quality register designed specifically for spine surgery, provides procedure-specific data for all hospitals performing ACDF and enables population-level assessment of analgesic use. Individual-level linkage of a complete national prescription dataset with a dedicated surgical quality register is uncommon in spine research and enhances the reliability of the exposure and outcome measures.

Several limitations merit consideration. Prescription records cannot verify actual medication use or the pattern of drug intake, which is an inherent limitation of purchase-based research. While prescription data are complete, patient-reported and surgeon-reported variables in FinSpine are not. Nonresponse bias has previously been evaluated and found not to distort clinical outcome estimates in ACDF cohorts within FinSpine or in a comparable Norwegian spine register, although missing data reduce the number of patients available for multivariable modeling.^40,41^ Currently, FinSpine lacks detailed information on concomitant pain conditions, psychiatric comorbidity, and broader socioeconomic factors, which may contribute to residual confounding. Accordingly, preoperative purchase rates should not be interpreted as directly proportional to cervical symptom severity (NDI or VAS), as purchases may reflect other indications and unmeasured behavioral and socioeconomic factors. Finally, for the subgroup of new strong-opioid users, the low event count limited statistical power, and the absence of independently associated factors should be interpreted cautiously.

## CONCLUSION

After ACDF for degenerative cervical spine disease, new sustained strong-opioid use was uncommon among previously strong-opioid naïve patients. In contrast, a substantial proportion of those who used opioids and gabapentinoids preoperatively continued to use them postoperatively. Long preoperative pain duration, smoking, and preoperative purchases of the same medication were independently associated with continued postoperative use, although the latter requires cautious interpretation due to potential confounding.

Nationwide prescription data provide an objective and complete measure of postoperative medication dispensing patterns and offer an endpoint that is not affected by nonresponse or missing data. Integrating prescription-based endpoints with established outcome parameters such as NDI, VAS, and return-to-work rates may offer a more comprehensive, quantifiable, and objective assessment of recovery in future studies.

Key Points

- In a nationwide FinSpine cohort, 4366 consecutive patients underwent ACDF for DCSD.
- Postoperatively, 16.5% used opioids and 15.6% gabapentinoids.
- Longer pain duration, smoking, higher NDI, and adverse working status predicted continued postoperative use.
- New postoperative strong-opioid use was observed in 2.2% of previously strong-opioid naïve patients.
- Preoperative use of the same drug was associated with postoperative use.

## Supplementary Material
