# Supplementary material for: Opioid and Neuropathic Pain Medication Use After ACDF for Degenerative Cervical Spine Disease—Nationwide FinSpine Register Study
Source: Spine (Phila Pa 1976). 2026 Apr 9;51(17):1223–32. doi: 10.1097/BRS.0000000000005705 (PMC13440540; doi:10.1097/BRS.0000000000005705)
Supplement: Supplementary file 1 [file brs-51-1223-s001.pdf]

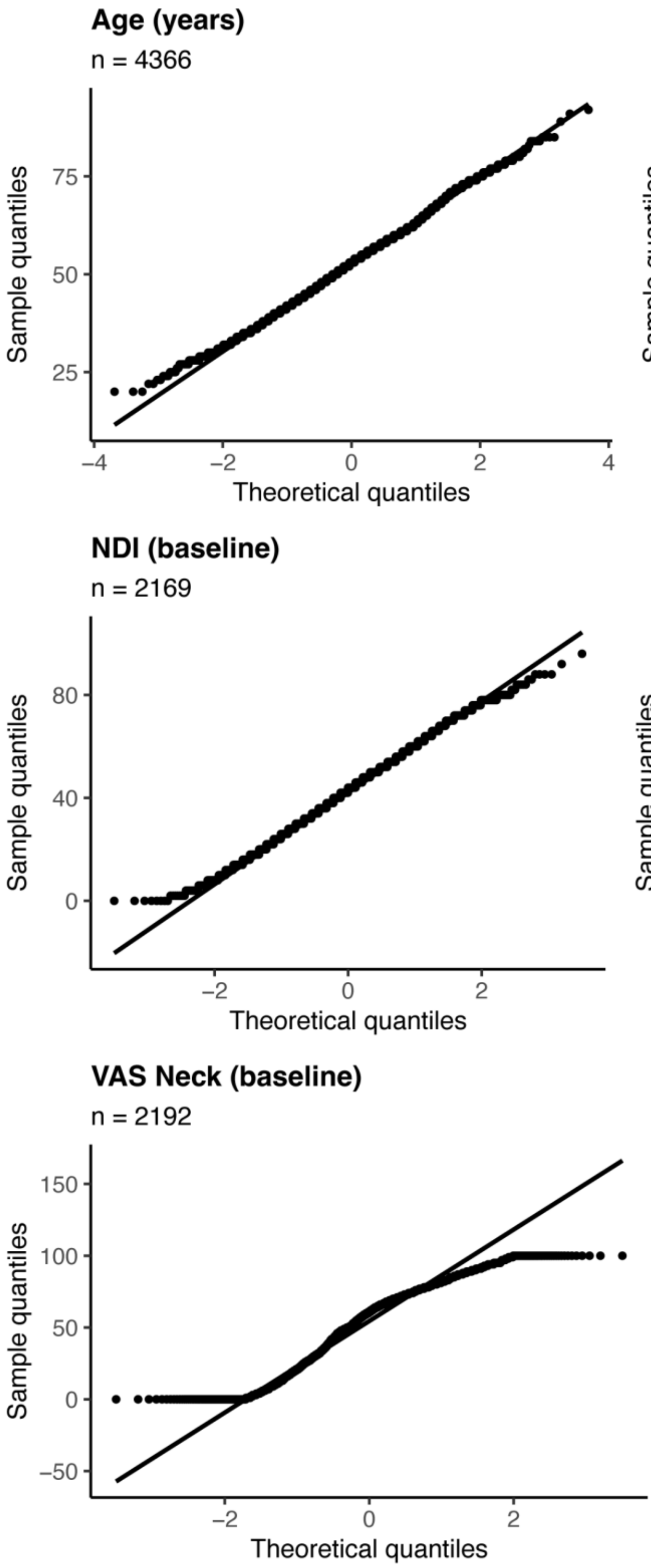

Supplementary Digital Content 1. Q–Q Plots for Baseline Continuous Variables

Normal Q–Q plots for age, BMI, NDI, VAS Arm pain and VAS Neck pain.
Plots illustrate distributional characteristics of baseline continuous variables used in descriptive group comparisons.
Abbreviations: BMI, body mass index; NDI,Neck Disability Index; VAS, visual analogue scale
