# Supplementary material for: Opioid and Neuropathic Pain Medication Use After ACDF for Degenerative Cervical Spine Disease—Nationwide FinSpine Register Study
Source: Spine (Phila Pa 1976). 2026 Apr 9;51(17):1223–32. doi: 10.1097/BRS.0000000000005705 (PMC13440540; doi:10.1097/BRS.0000000000005705)
Supplement: Supplementary file 2 [file brs-51-1223-s002.pdf]

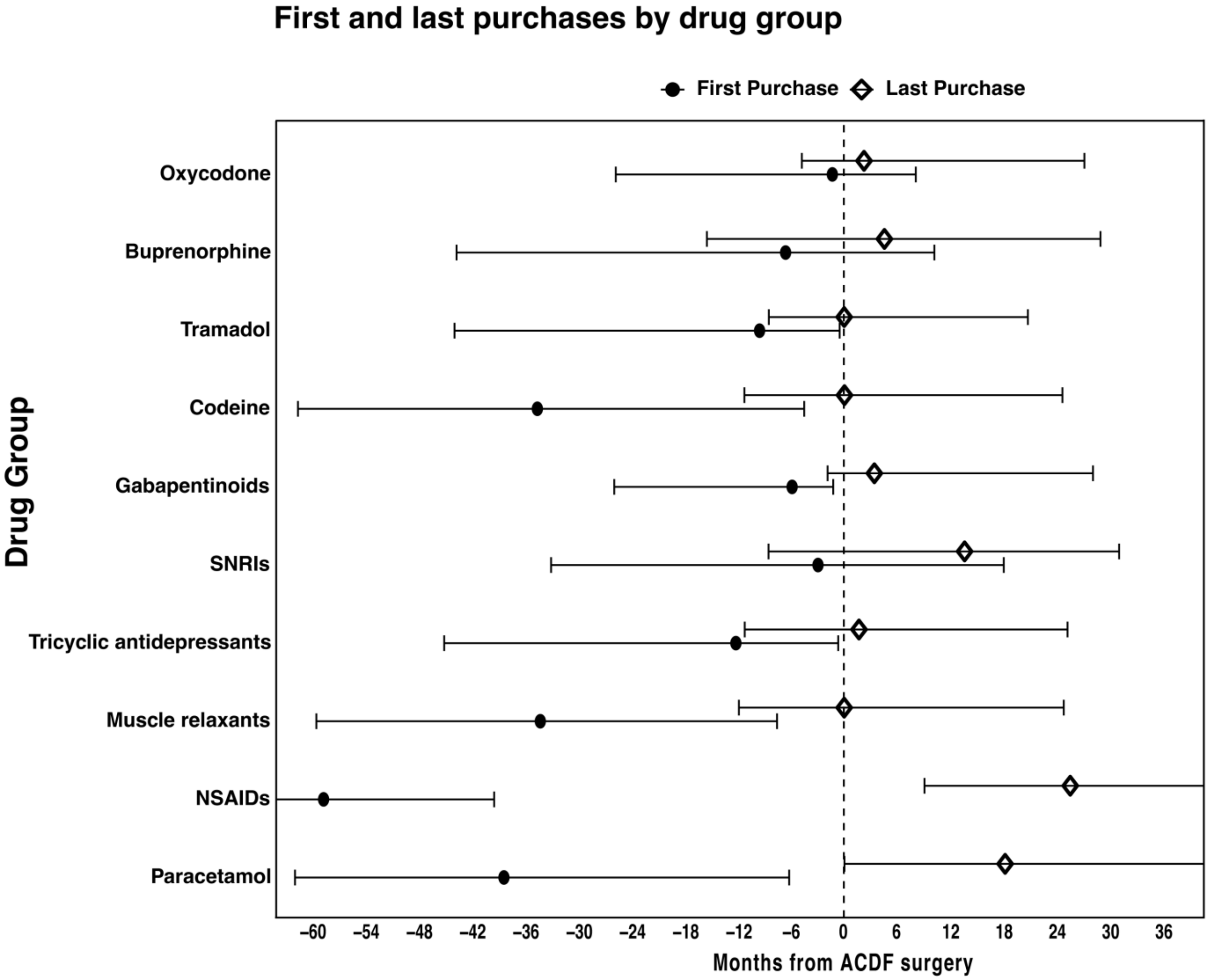

Supplementary Digital Content 3. Median first and last purchases for each drug category in the FinSpine study population in relation to ACDF; Negative values indicate preoperative timing. Horizontal bars indicate IQRs; intervals extending beyond the plotted range are truncated at the axis limits. Abbreviations: SNRIs, serotonin–noradrenaline reuptake inhibitors; NSAIDs, non-steroidal anti-inflammatory drugs.
