# Supplementary material for: Opioid and Neuropathic Pain Medication Use After ACDF for Degenerative Cervical Spine Disease—Nationwide FinSpine Register Study
Source: Spine (Phila Pa 1976). 2026 Apr 9;51(17):1223–32. doi: 10.1097/BRS.0000000000005705 (PMC13440540; doi:10.1097/BRS.0000000000005705)
Supplement: Supplementary file 4 [file brs-51-1223-s004.docx]

| **Model** | **N** | **Events** | **AUC** | **Brier**  **Score** | **Calibration Intercept** | **Calibration Slope** |
| --- | --- | --- | --- | --- | --- | --- |
| Oxycodone | 1770 | 44 | 0.854 | 0.021 | -0.232 | 0.850 |
| Buprenorphine | 1770 | 28 | 0.828 | 0.016 | -0.399 | 0.764 |
| Tramadol | 1770 | 119 | 0.825 | 0.053 | -0.093 | 0.882 |
| Codeine | 1770 | 141 | 0.777 | 0.065 | -0.081 | 0.884 |
| Gabapentinoids | 1770 | 267 | 0.787 | 0.108 | -0.027 | 0.916 |
| New strong-opioid users | 1770 | 33 | 0.710 | 0.019 | -0.335 | 0.698 |

**Table, Supplementary Digital Content 2.** Internally validated performance of multivariable models for repeated postoperative purchases and new strong-opioid user status.

Values are optimism-corrected using bootstrap internal validation (500 resamples) for multivariable logistic regression models using Firth’s penalised likelihood. N represents the total number of complete-case observations included in each model. Events represents the number of patients with the model outcome.
